# From a Lived Event to Its Autobiographical Memory: An Ecological Study Using Wearable Camera in Schizophrenia

**DOI:** 10.3389/fpsyt.2019.00699

**Published:** 2019-10-04

**Authors:** Mélissa C. Allé, Anne Giersch, Jevita Potheegadoo, Nicolas Meyer, Jean-Marie Danion, Fabrice Berna

**Affiliations:** ^1^Inserm U1114 - Cognitive Neuropsychology and Pathophysiology of Schizophrenia, Strasbourg, France; ^2^FMTS: Fédération de Médcine Translationnelle de Strasbourg, University of Strasbourg, Strasbourg, France; ^3^University Hospital of Strasbourg, Strasbourg, France

**Keywords:** autobiographical memory, event segmentation, temporal organization, schizophrenia, wearable camera

## Abstract

Cognitive disorders are considered as a core symptom of schizophrenia. Importantly, episodic autobiographical memory deficits are strongly related to patients’ social dysfunction. Although the cognitive mechanisms underlying autobiographical memory deficit are highly important to open the door for specific cognitive remediation, they are yet to be understood. The present study focused on event segmentation to check to which extent possible impairments in temporal ordering and segmenting in patients hinder memories construction. Twenty-seven patients with schizophrenia and 27 matched controls took part in an outdoor circuit while wearing a wearable camera. A week later, their memory and the temporal organization of this event have been assessed. Results showed that patients, compared with control participants, reported a reduced amount of details, especially less actions with interaction related to the event. Contrary to our initial hypotheses, event segmentation abilities in patients were not affected. The relationship between event segmentation and memory is discussed.

## Introduction

Cognitive disorders, comprising processing speed, episodic memory, working memory, and executive functioning, are considered as a core symptom of schizophrenia (1), as they play an important role in the etiology, course, and outcome of the illness (2, 3). Indeed, these cognitive deficits are linked to poorer functioning and quality of life (2, 4). Importantly, deficits of episodic memory for personal events have been shown to be related to patients’ social dysfunction, over clinical symptoms and other cognitive deficits (5). In addition, the ability to recall a particular type of episodic memory (self-defining memories) is associated with engagement in structured activity in people with first episode psychosis (6). Hence, understanding patients’ episodic memory deficits in daily life may help to better grasp factors responsible for their social dysfunction. In addition, episodic autobiographical memory sustains a sense of self-continuity across time (7), and those memory alterations might be one of the mechanisms accounting for patients’ self disorders (8, 9).

Episodic memory is defined as the ability to recall specific episodes integrated in a temporospatial context and is associated with conscious recollection (10). According to Conway’s model of the (11), episodic memory retains highly specific sensory and perceptual details of recent experiences over retention intervals measured in minutes and hours. Episodic memories do not endure unless they are highly significant for the self and become linked to autobiographical memory structures (11).

In most studies, episodic memory dysfunctions in schizophrenia have been highlighted using standard laboratory recall and recognition tasks, where lists of verbal materials are presented and participants later asked to recall or recognize them. Episodic memory has been shown to be particularly affected in schizophrenia (12, 13), and these impairments appeared to be largely due to defective strategic processes at encoding and, more precisely, to default self-initiation strategies observed in patients with schizophrenia (14, 15).

Moreover, the ability to recall personally experienced past events, known as autobiographical memory, has also been shown to be severely impaired in schizophrenia (16, 17,). Patients with schizophrenia recalled less information and fewer details from personal past events in comparison with control participants (19). Their memories are also less specific, less vivid, and less associated, with a feeling of reliving (17) and lacking contextual information (13, 17). Overall, these impairments have been suggested to relate to patients’ executive dysfunction at retrieval (20). So far, all the investigations of autobiographical memory have been retrospective, referring to the memory of events that have taken place months or years before testing, such that it is impossible to control the nature of the encoded information. Although a few studies suggested that encoding deficits also participate to autobiographical episodic memory impairments in schizophrenia (13, 20, 21), evidence remained indirect. Hence, understanding the cognitive mechanisms underlying autobiographical memory deficits observed in schizophrenia and whether encoding, retrieval, or both are impaired are highly important, as it may open the door for specific cognitive remediation therapies. In the present study, we focus on event segmentation to check to which extent possible impairments in temporal ordering and segmenting (22) hinder memory construction.

The event segmentation theory postulates that segmenting an ongoing activity into meaningful events is a core component of ongoing perception and affects the way people later remember the whole episode (23). According to this theory, everyday life is generally perceived as a continuous sequence of different actions that our brain automatically and unconsciously split into smaller components (or segments), to organize the continuous flow of events (24, 25). The segments result from the perceptual segmentation and form the basis of memory and learning. Findings indicated that individuals who are better at segmenting an activity into events in a coherent manner are better at remembering it later (26). Evidence indicates that the brain and mind track features of one’s environment, and when a salient feature changes unpredictably, an event boundary is perceived (25). These features are either sensory (such as color, sound, or movement) or conceptual (such as cause and effect interactions, and goals). The authors of the event segmentation theory distinguish two levels of segmentation, based on these features. First, sensory features are thought to generate fine segmentation, primarily a bottom–up process. Second, segmentation based on conceptual features relies on top–down stored knowledge and leads to a goal-directed coarse segmentation, where event sequences are split into meaningful units. Event segmentation is considered as one of the fundamental mechanisms by which human cognition collects, organizes, encodes, and stores the large variety of information extracted from the environment (25).

Event segmentation process is sustained by elementary cognitive functions such as working memory, updating, and attention (23), which are described as severely impaired in schizophrenia (1). In addition, event segmentation requires a good understanding of event thematic organization and a flowing temporal perception—two properties actually impaired in patients with schizophrenia. First, difficulties to thematically understand and organize non-autobiographical stories have been observed in schizophrenia (27), and the thematic coherence of patients’ autobiographical narratives is also decreased (16, 28). Second, timing disorders have been described at various levels in schizophrenia, from asynchrony detection and (29–31) temporal order judgment deficits at very short scales of time (millisecond) (32) to impairments of working memory and episodic memory temporal components (33–35). In addition, patients display difficulties to estimate the temporal distance of personally experienced past events (36), and the narratives of patients’ personal life are also temporally disorganized (37,). Nevertheless, studies also showed that patients are able to use temporal cues appropriately to create clusters of autobiographical memories (38). It has been previously shown that patients with schizophrenia have difficulties in segmenting movies of daily life activities (39).

All together, the literature suggested that event segmentation might be impaired in schizophrenia and related to patients’ autobiographical memory deficits. Hence, this study aimed at exploring autobiographical memory recollection and event segmentation of a recent and experimentally controlled life event. The relationships between the different levels of cognitive processes were also examined.

For this purpose, we designed a new protocol investigating autobiographical episodic memory with a prospective approach. To the best of our knowledge, it is the first time that autobiographical memory has been assessed with such an ecological study design in schizophrenia. Participants took part to a walk in the city center, wearing a small camera around their neck. A complex event, similar to daily life events, has thus been created to investigate as precisely as possible the autobiographical memory of this event. Wearable cameras were used to better test memory recall from what happened during the event (40). The prospective approach enabled us to control the event characteristics and be sure that differences between patients and controls in recall could not be explained by initial event differences.

Based on the earlier mentioned literature, we hypothesized that autobiographical memory of patients with schizophrenia would be impaired for this recent event, in terms of vividness and chronological organization, and that event segmentation would be impaired in patients, in comparison with control participants. Finally, we hypothesized that basic cognitive deficits would be related to both event segmentation and autobiographical memory impairments in schizophrenia.

## Material and Method

### Participants

Twenty-seven stabilized outpatients (seven women) were recruited from the Psychiatric Department of Strasbourg’s University Hospital, all fulfilling the Diagnostic and Statistical Manual of Mental Disorders, 5th Edition diagnosis criteria for schizophrenia. Patients with a current major depressive episode (scores higher than 6 on the Calgary Depression Schizophrenia Scale) (41) were excluded. All patients except one were receiving long-term antipsychotic treatment. Patients treated with benzodiazepines were not included. The control group included 27 healthy participants (7 women). Patients and controls had neither current substance abuse nor a history of traumatic brain injury, epilepsy, or other neurological disorders. The two groups did not differ significantly in terms of age, level of schooling, or premorbid IQ (see **Table 1**).

**Table 1:** Demographic, clinical, and initial assessment measures of patients with schizophrenia and controls

	Patients with schizophrenia (n = 27)		Control participants (n =27)		t-test		
	Mean	*(SD)*	Mean	(*SD*)	t	*p-va*lue	*Effect size (d)*
Age (years)	37.96	(9.25)	38.89	(9.69)	-0.36	.72	–0.10
Years of schooling	12.93	(2.02)	13.11	(1.93)	-0.34	.73	–0.09
BDI	–	–	2.18	(2.75)	–	–	–
CDSS	1.08	(1.38)	–	–	–	–	–
Onset of the illness (years)	21.67	(5.29)	–	–	–	–	–
Duration of illness (years)	12.2	(7.45)	–	–	–	–	–
PANSS (total score)	63.65	(16.50)	–	–	–	–	–
- Positive symptoms	15.69	(4.65)	–	–	–	–	–
- Negative symptoms	18.27	(7.55)	–	–	–	–	–
- General psychopathology	30.38	(9.38)	–	–	–	–	–
**Cognitive Assessment**							
f-NART (premorbid IQ)	108.44	(6.95)	109.52	(6.80)	–0.57	.57	–0.16
Verbal fluency* (semantic)	–0.36	(0.84)	0.17	(0.71)	–2.48	.02	–0.27
Verbal fluency* (phonemic)	–0.03	(0.80)	0.15	(0.94)	–0.75	.46	–0.13
Shifting score* (TMT B-A)	0.05	(0.91)	0.01	(0.75)	0.19	.85	0.05
Updating^¤^ (N-back)	7.03	(2.71)	5.23	(2.05)	2.73	.009	–0.88
Spatial working memory*	9.81	(1.96)	11.18	(2.42)	–2.27	.03	–0.57
**Temporal order judgement**							
100 ms (Errors proportion)	0.07	(0.07)	0.03	(0.05)	2.14	.04	–0.80
500 ms (Errors proportion)	0.06	(0.07)	0.02	(0.03)	2.55	.01	–1.33
**Non-autobiographical stories sequencing**							
Behavioral stories (Errors score)	8.63	(4.11)	7.11	(3.43)	1.47	.15	–0.44
ToM stories (Errors score)	14.67	(8.15)	11.78	(7.81)	1.33	.19	–0.37
Mean Errors score	11.65	(4.96)	9.44	(4.98)	1.63	.11	–0.44

BDI, Beck Depression Inventory; CDSS, Calgary Depression Scale for Schizophrenia; PANSS, Positive and Negative Symptoms Scale; f-NART, French National Adult Reading Test; IQ, intellectual quotient; TMT, Trail Making Test.

*z-scores.

number of errors.

A third group (n = 12), called the reference group, was also included (mean age = 27.91 years old; mean level of schooling = 16.5 years) in order to calibrate the tests used to assess the recall of the outdoor circuit. These participants were not matched with the two other groups, but their results were used as references to be compared with both patient and control groups (see *Memory Tasks* section for more explanations).

### Initial Assessments

#### Clinical Assessment

The severity of patients’ symptoms was assessed by the Positive and Negative Syndrome Scale (42). All patients were clinically assessed for depression, with the Calgary Depression Scale for Schizophrenia (41), and control participants completed the Beck Depression Inventory (43).

#### Neuropsychological Assessment

A series of tests were selected to assess specific cognitive functions and skills known to be involved in event segmentation (25). Specific executive functions were assessed, using the Trail-Making Test (TMT-Part A and B) (44) for mental flexibility, the semantic and phonologic verbal fluency tasks (45) for strategic retrieval of information in memory, the N-back (N-3) (46) for updating process, and the Corsi’s Cube (47) for visuo-spatial memory.

#### Temporal Order Judgement Task

A computerized task was used to assess participants’ ability to identify the temporal order of two events at very small time scale (48). A central fixation point was first presented on a black screen, where it remained for 500 ms. Then, frames appeared simultaneously. After a further delay of 100 ms, two targets filled the inside of the frames successively. We manipulated the stimulus onset asynchrony between the apparition of the first and the second targets. In order to check whether patients were able to detect temporal order for explicit and easily detectable stimuli, the stimulus onset asynchrony was 100 ms for 160 trials and 500 ms for 20 trials. The number of judgment errors was counted for both conditions.

#### Non-Autobiographical Stories Sequencing Task

Participants were presented with 16 stories depicted in five-card picture sequences using a simple black-and-white cartoon style (27). We used two types of sequences. (a) *Behavioral stories:* Eight sequences of purposive actions performed by a single character acting within a narrow spatio-temporal context and using familiar objects or tools (*make a toast, have a soft drink, go to bed, wash one’s hands, brush one’s teeth, warm up milk, phone, get dressed*). (b) *Theory of Mind stories*: Eight sequences of false-belief stories describing an event in which one of the characters is unaware of what is happening (*a boy takes the man’s newspaper; a boy takes the girl’s pizza, a man steals the girl’s doll; a girl steals the boy’s marbles; a man takes the boy’s shovel; a girl takes the boy’s head of apples; a girl takes the boy’s pail; a girl takes the boy’s car*). In order to arrange these stories chronologically, participants must infer that this character acts according to his/her own false belief about the situation. The total number of incorrect positions was counted for each story, giving us an error score.

### Procedure for Autobiographical Memory Assessment

The experiment consisted of two phases: first, a walk on the city center, where participants accompanied the experimenter through the city and performed a series of activities at different locations and, second, a retrieval phase to assess the memory of the walk.

#### Walk in the City Center

**Figure 1:**
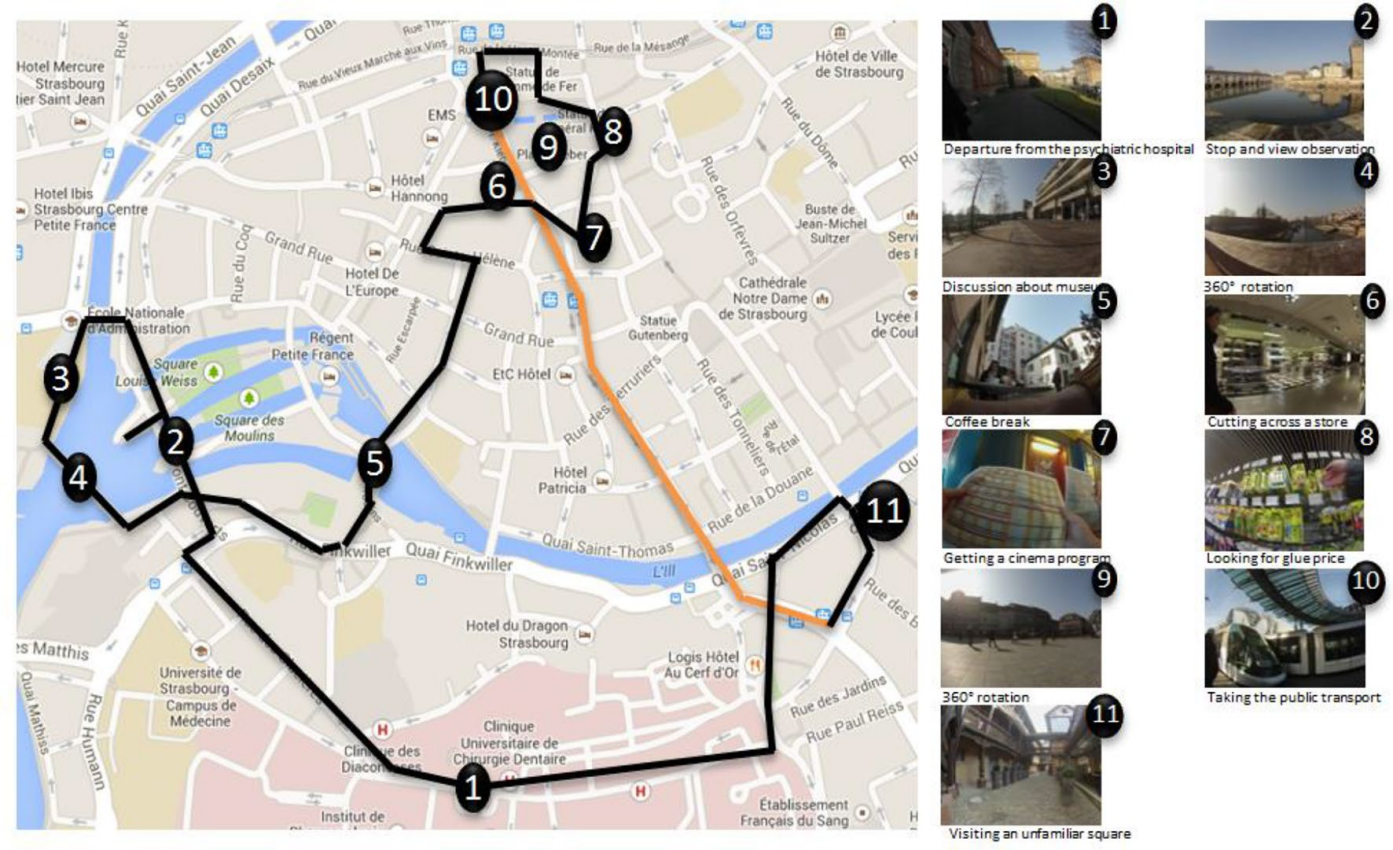
Overview of the walk on the city center of Strasbourg (France). The numbers represent the locations in which activities were performed, black lines represent the walk between each activity, and the orange line represents public transport use. For each numbers, an example of pictures taken by the wearable camera during the activity is provided. Map data copyright: Google, 2017.

Participants took part to a 1-h outdoor circuit created for the study (see **Figure 1**). This event was composed of different daily life activities (e.g., going to the supermarket, using public transport, or looking for a cinema program) and also rarer events (e.g., visiting an unfamiliar square). The circuit was entirely guided by the experimenter in order to replicate exactly the same walk with each participant. Before starting the walk, the following instructions were given to the participant: *“We are going for a walk in the city center. The circuit is composed of different daily life activities. Everything has been planned in advance, we cannot change the direction during the pathway. Please pay attention to what happens, we will perform memory tests about this circuit next week”*.

In order to record each participant’s pathway, a small camera (GoPro^®^ Hero3) was worn by every participant, hung with a lanyard around their neck. This device was programmed to automatically take fish-eye pictures every 10 s during the whole circuit.

#### Memory Tasks

One week after the circuit, participants came back to complete the memory tests.

##### Memory Chronological Organization Task

Three series of 12 personal pictures, taken during the initial event, were presented to the participant on a computer screen. These pictures were selected to represent exactly the same moment of the outdoor circuit for each participant and were placed in the same nonchronological order. Participants were asked to chronologically order pictures according to what originally happened during the pathway. The chronological organization was assessed through two different scores. First, the chronological score corresponded to the mean number of pictures correctly ordered within each series. Second, the deviation score corresponded to the mean number of pictures deviating from its correct position.

##### Event Segmentation Task Procedure

The event segmentation task was adapted from a previous study conducted by Zacks and colleagues (49). Participants were presented a slideshow on a computer screen (1 picture per 0.75 s), containing every picture taken during the initial event. While watching the slideshow, they were asked to stop it (pressing the spacebar) when they perceived a boundary between two events they had lived (i.e., switching from one event to another one). In other words, participants had to press the spacebar each time an event ended and/or another one started. In this study, contrary to Zacks’ protocol (24), participants were asked to segment the slideshow according to their own internal criteria, no particular instructions regarding the grain of the segmentation was provided. This choice was motivated by the need to make the protocol shorter and less demanding for patients and to assess which criteria participants spontaneously used to segment the circuit. Participants were instructed to identify temporal boundaries as accurately as possible and were allowed to look at the video backward to mark the boundary. Each time they pressed the spacebar, they were asked to explain on which criterion they based their segmentation. Responses of the participants were given orally and recorded for later analysis.

###### Scoring

The number of boundaries, representing the number of stops during the slideshow display, was recorded. In addition, two categories of boundaries were considered, either based on environment perceptual changes (fine segmentation) or on action realized (coarse segmentation). Two independent raters (blinded to the hypothesis) determined the category (perceptual changes or action) of each boundary, based on participant’s own criterion description reported during the segmentation (κ = 0.77). The total number of boundaries for each category was considered.

In addition, two scores were created to assess segmentation abilities.

First, the boundaries typicality score reflected the extent to which a participant (from patient and control groups) segmented the circuit in agreement with a majority of persons. We considered the 20 most frequent boundaries of the reference group and attributed a score to each boundary, based on its frequency within the group^1^. For each participant, the mean of each boundary score was calculated.

Second, the temporal accuracy score defined the temporal deviation between a participant’s boundary and the mean segmentation of the reference group on the same prototypical boundary. Slideshows were unique for each participant and thus differed with regard to their temporality. Therefore, we performed a normalization of the data in order to compare temporal deviation across participants. Importantly, the temporal accuracy score was calculated only for the 30 most frequent boundaries, called “prototypical boundaries” of the reference group. A high score corresponds to an important temporal deviation from the prototypical boundaries.

##### The Free Recall Task

Participants were asked to freely recall everything that happened during the walk on the city, in as much detail as possible. In comparison with a list of items (i.e., movements, actions, perceptual details, etc.), based on the free recall results of the reference group, the number of details and actions correctly reported by participants was recorded by the experimenter, and the total number of items was considered for analyses.

In addition, verbal reports were recorded for two thirds of the participants (n = 34), using a digital audio record and verbatim transcribed. Verbal descriptions were then analyzed according to Jeunehomme and colleagues scoring method (50), which enables a deeper exploration of participants free recall. Verbal descriptions were first divided into experience units, which correspond to a particular moment of experience (“I got out the store and turned left”; “I saw a man and his dog walking down the street”). Two independent raters, both blinded to the hypothesis, divided all the narratives into experience units, and Intraclass Correlation Coefficients (ICCs) showed a strong agreement between them (r_ICC_ = 0.88). The second step consisted of analyzing the content of each experience unit that includes one or several pieces of information. Each information is called unit component and can be classified according to different categories: person, object, thought, action with interaction, and spatial movement (see **Table 2** for descriptions and examples). One experience unit can include several components. Thirty percent of the experience units, randomly selected, were rated by two independent raters. ICC showed moderate to strong agreement regarding the number of components described per experience unit (objects = .71; persons = .62; thoughts = 0.74; actions with interaction = .84; spatial movements = .93; perceptual details = .64; spatial details = .52; comment = .97).

**Table 2:** Descriptions and examples of scored experience unit components

Component categories	Description and examples
Person	Description of one or more person(s), with no description of interacting with this/these person(s) (if an interaction is described, the component was classified as “action with interaction”)
Object	Description of an object or aspect of the external environment with no description of interaction with this object
Thought	Description of a thought, mental state, or judgement
Action with Interaction	Description of an action performed by the participant involving a direct interaction with an object or a person
Spatial Movement	Description of a movement of the body in the environment
Perceptual Detail	Description of a sensory detail about an object or a person (i.e., a texture, shape, or color), or of an internal sensation
Spatial Detail	Description of a detail replacing the spatial context of an object or a person
Comment	Explanations or clarifications that do not in themselves describe moments of experience

From “Temporal compression in episodic memory for real-life events,” by O. Jeunehomme, A. Folville, D. Stawarczyk, M. Van der Linden, and A. d’Argembeau, 2018, Memory, 25:1–12. Copyright by Olivier Jeunehomme. Reprinted with permission.

### Statistical Analyses

Between-group comparisons of clinical, neuropsychological, and autobiographical memory measures were performed using Student t-tests. To analyze the segmentation task, an analysis of variance was also performed with the group (patient group vs. control group) as a between-subject factor and category of boundaries (perceptual vs. action) as a within-subject factor. Finally, Pearson correlations were calculated between 1) neuropsychological, temporal order judgement and non-autobiographical stories sequencing measures and 2) autobiographical memory measures (chronological organization, event segmentation, and free recall).

## Results

### Initial Assessments

#### Neuropsychological Assessment

Patients and controls did not differ in terms of IQ. Patients’ executive functioning was significantly lower than that of controls for categorical verbal fluency, updating, and visuo-spatial memory, whereas the phonemic verbal fluency and the mental flexibility score of the TMT (TMT B-A) did not differ significantly between groups (see **Table 1**).

#### Temporal Order Judgment Task

Patients with schizophrenia made significantly more errors than controls when asked to judge the temporal order between two stimuli appearing successively, regardless of the delay (100 or 500 ms) between each stimulus (see **Table 1**).

#### Non-Autobiographical Stories Sequencing Task

Patients made more errors than controls in ordering both behavioral and theory of mind stories, yet these differences were not significant (see **Table 1**).

### Walk in the City Center

All participants fully completed the outdoor circuit. No significant difference was observed between patients and controls regarding the mean duration of the pathway (*p* = .27). The extent to which participants know the city center in which the outdoor circuit took place has been controlled through a questionnaire and was equivalent across group (data not shown).

### Autobiographical Memory Tasks

#### The Chronological Organization Task.

Patients’ performances did not differ from those of controls on the three scores assessing the chronological organization of the memory (see **Table 3**).

**Table 3:** Mean scores of patients with schizophrenia and control participants for autobiographical memory assessment.

	Patients with schizophrenia (n = 27)		Control participants (n = 27)		t-test		
	Mean	*(SD)*	Mean	*(SD)*	*t*	*p-value*	Effect size *(d)*
**Chronological organization task**							
Chronological Score	7.41	(2.70)	7.42	(2.14)	-0.03	.98	-0.005
Deviation Score	1.47	(0.84)	1.48	(0.80)	-0.34	.96	-0.01
**Event segmentation task**							
Temporal accuracy score	1.61	(0.61)	1.66	(0.89)	-0.14	.89	-0.06
Boundaries typicality score	6.09	(1.50)	6.36	(2.08)	-0.53	.60	-0.13
**Free recall task**							
**- All participants**							
Number of items recalled	25.17	(7.67)	31.11	(5.33)	-3.24	.002	-1.11
**- Subgroup of participants (see Methods)**							
Number of experience units^a^	22.75	(10.43)	27.94	(6.92)	-2.01	.051	-0.60
%Person	1.26	(2.06)	1.97	(3.00)	-0.78	.44	-0.28
%Object	6.78	(7.23)	4.97	(4.31)	0.84	.40	0.31
%Thought	2.05	(2.69)	3.17	(3.99)	-0.94	.36	-0.33
%Action with Interaction	17.10	(9.27)	29.42	(10.81)	-3.47	.002	-1.23
%Spatial Movement	87.07	(22.02)	95.49	(16.27)	-1.21	.23	-0.44
%Perceptual Detail	2.29	(4.05)	4.13	(5.18)	-1.12	.27	-0.40
%Spatial Detail	9.92	(9.99)	12.12	(9.16)	-0.64	.52	-0.23
%Comment	9.61	(2.55)	7.68	(8.51)	0.28	.78	0.35

SD, standard deviation; ToM, theory of mind.

a One unit can be composed of several unit components.

#### The Event Segmentation Task

**Figure 2:**
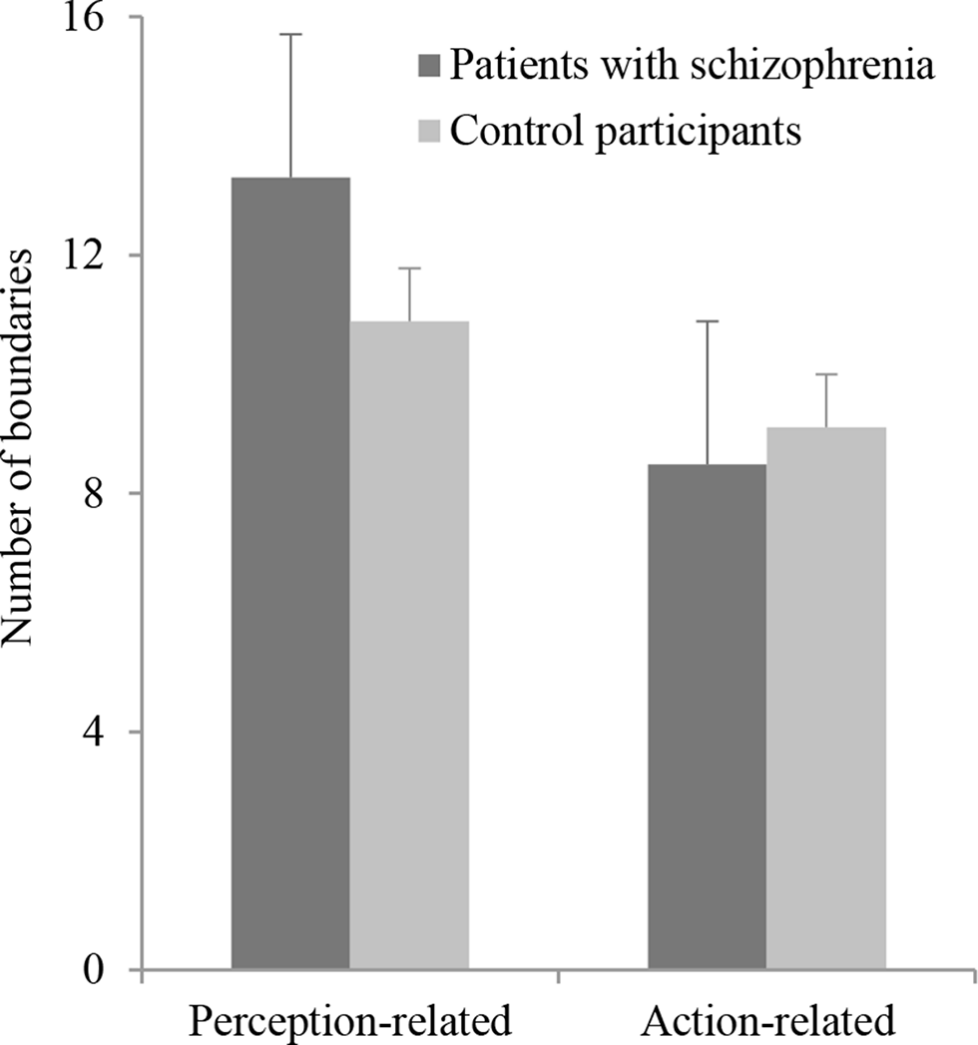
Mean number of boundaries for perception-related and action-related segmentation, in both patient and control groups. Error bars represent the standard errors of the means.

A two-way analysis of variance showed a significant effect of the category of boundaries, *F*(1,52) = 11.13; *p* = 0.002, with the number of perceptual boundaries being higher than the number of action boundaries. The number of boundaries identified by the participants did not differ between groups, *F*(1,52) = 0.62; *p* = 0.44, and the interaction between groups and boundaries categories was not significant, F(1,52) = 2.36; p = 0.13 (see **Figure 2**). Besides, none of the other scores depicting segmentation abilities (the boundaries typicality and the temporal accuracy scores) significantly differed between patients and controls (see **Table 3**).

#### The Free Recall Task

First, when considering the whole group of participants, patients with schizophrenia recalled significantly fewer items than controls (see **Table 3**). Second, when analyzing only the verbatim narration, the number of experience units was also lower in the patient group. Finally, the analysis of experience units’ content showed that patients recalled twice less actions with interaction compared with controls, all other components being equally recalled.

### Correlation Analyses

We conducted exploratory correlation analyses to examine the relationship between autobiographical memory characteristics and cognitive measures (see **Table 4**). In the patient group, significant positive correlations were found between the temporal accuracy scores (event segmentation task) and 1) errors rates in the temporal order judgement task (for both 100 and 500 ms), and 2) working memory scores. Hence, patients who made more errors in the temporal order judgement task or those with lower working memory functioning displayed lower temporal accuracy in the segmentation task. Moreover, the boundaries typicality score was negatively correlated with the total number of boundaries in patients. It means that the more patients segmented the slideshow, the less typical were their boundaries. Finally, the boundaries typicality score was significantly correlated with the number of experience units recalled in the free recall task. Thus, patients who segmented typical boundaries in the event segmentation task later remembered more experience units. No significant correlation was found between autobiographical memory measures and symptoms severity in the patient group.

**Table 4:** Correlation analyses between cognitive and autobiographical memory measures.

Patients Upper Controls Lower	The chronological organization task		The event segmentation task			Free recall task	
	Chronological Score	Deviation Score	Number of boundaries	Temporal accuracy score	Boundaries typicality score	Number of items	Number of experience units
Temporal order judgement 100 ms (errors)	0.11 –0.42*	-0.07 –0.07	0.05 –0.17	0.51** 0.01	0.15 0.11	–0.07 0.07	0.46 –0.14
Temporal order judgement 500 ms (errors)	–0.09 –0.05	0.09 0.22	–0.27 0.08	0.46* 0.04	–0.08 0.10	–0.20 –0.02	–0.33 –0.17
Spatial Working Memory	0.13 0.09	–0.12 –0.35	–0.10 –0.24	-0.41* 0.13	0.16 –0.01	0.14 0.08	0.22 –0.26
Updating	–0.10 –0.32	0.26 0.11	0.03 0.10	0.28 0.24	–0.07 –0.28	0.28 –0.07	0.09 0.16
Phonologic Fluency	–0.09 0.05	0.11 0.01	0.10 0.09	-0.36 0.01	–0.01 –0.03	0.21 –0.17	0.17 –0.38
Semantic Fluency	–0.17 –0.25	0.01 –0.25	0.18 –0.03	-0.06 -0.11	–0.07 0.23	–0.07 0.12	–0.29 –0.10
Non-autobiographical stories sequencing (errors)	–0.33 0.03	0.29 0.30	–0.34 0.19	-0.08 0.13	0.02 –0.15	–0.41* –0.17	0.02 –0.04
**The chronological organization task**							
Chronological Score	–	–0.92*** –0.57***	0.27 –0.44*	0.11 –0.06	–0.15 0.04	0.34 0.35	0.19 0.26
Deviation Score		–	–0.33 0.33	–0.17 0.20	0.27 –0.17	–0.21 –0.44*	–0.10 –0.02
**The event segmentation task**							
Number of boundaries			–	0.19 0.07	–0.60*** –0.26	0.14 –0.28	0.12 –0.19
Temporal accuracy score				–	–0.01 –0.63***	–0.18 –0.04	–0.14 0.26
Boundaries typicality score					–	0.18 0.07	0.53* –0.20

In the control group, significant correlations were observed between the chronological score and 1) the errors rate of temporal order judgement (100 ms), and 2) the number of boundaries defined by the participants. Participants with fewer errors on the temporal order judgement task had higher chronological score in the autobiographical memory task. Moreover, in the event segmentation task, the boundaries typicality score was negatively correlated with the temporal accuracy scores, meaning that the more typical the boundaries were, the more temporally accurate was their segmentation. Finally, the amount of items recalled was negatively correlated with the deviation score of the chronological organization.

It is, however, worth stressing that when correlations were adjusted with Bonferroni correction, none of them remained significant. However, we decided to present the correlation analyses without Bonferroni correction as an exploratory work.

## Discussion

The present exploratory study sought for the first time to investigate the memory of a particular single event experienced 1 week earlier and the contribution of event segmentation abilities to the memory of this event in a group of patients with schizophrenia. The originality and strength of the present study are the ecological nature of this new protocol that contrasts with both the standard episodic memory tests (usually based on word lists or short third-person stories) and the usual autobiographical memory tasks (which focus on the collection of remote memories through interviews, without any control on the initial event). Our results showed that patients, compared with control participants, reported a reduced amount of details and less actions with interaction related to the event. Contrary to our initial hypotheses, event segmentation abilities were not affected in patients.

### Event Segmentation of the Memory

Results showed that patients with schizophrenia spontaneously segmented the initial event similarly to controls. In fact, neither the number nor the characteristics of the identified events’ boundaries differ between groups, meaning that the criteria used by participants to identify event boundaries (perception versus action) and the typicality of boundaries were roughly similar. These results are in opposition with those shown by Zalla and collaborators (39). In this previous study, participants were asked to segment small movies of daily life activities. Patients with schizophrenia had defined fewer correct boundaries for large events (sustained by goal-directed actions) but had equal number of correct small events (based on perceptual changes), reflecting patients’ difficulties to identify and segment goal-directed events. Although our results showed a trend in the same direction, we did not observe significant interaction between group and type of boundaries and did not confirm previous results with our material. Furthermore, no difference was observed between patients and controls regarding the temporal accuracy of the segmentation, again in opposition to what was previously observed (39). It is important to emphasize the methodological differences between the protocol of Zalla et al. and ours that may explain the discrepancy of the results observed between both studies. First, Zalla and colleagues’ material consisted of short movies of nonpersonal events encoded a few minutes before performing the segmentation task. This is in fact much different from the single 1-h trip that our participants personally experienced, then segmented 1 week later. Moreover, contrary to the continuous movies used by Zalla and colleagues, our participants segmented the past-lived event based on a pictures’ slideshow. The slideshow represents a discontinued flow of information that might have prevented us from precisely assessing participants’ temporal accuracy during segmentation.

Correlation analyses may help to better understand the processes involved in event segmentation. In the control group, we observed that the more typical a boundary was, the more temporally accurate the segmentation was, meaning that a typical event was more easily detected and identified than a rarer or nontypical event. It suggests that prototypical events benefited from more robust event models (i.e., somaticized representations of prototypical events stored in memory and used to predict ongoing perception) (25), enhancing events prediction and thus improving segmentation accuracy. In the patient group, this correlation was not observed, and in contrast, the higher the typicality of the boundaries, the lower the number of boundaries segmented. Moreover, the temporal accuracy of the event segmentation was related to patients’ cognitive skills, namely, basic temporal order judgement (ms scale) and working memory performances. Thus, the ability to discriminate the temporal order of two very simple events (2 squares) seemed related to the ability to accurately segment the sequence of information from a more complex event (the walk in the city center). Moreover, the relationship between temporal accuracy of event segmentation and working memory is in line with theoretical models stating that working memory enables to focus attention on the ongoing action, to keep it in short-term memory (51) and to better delineate action boundaries. Surprisingly, whereas these two cognitive processes (working memory and temporal order judgment) were impaired in the patient group, the temporal accuracy of event segmentation did not differ between the two groups. One may thus hypothesize that other cognitive processes in patients sustain or even alleviate patients’ capacity to accurately segment event in time. In fact, according to Zacks’ theory (23), event segmentation is sustained first by various cognitive functions but also by event models. These semantic representations of typical events provide knowledge on how an event is typically constructed and enable to anticipate the sequence of actions composing the stream of perception. Previous studies have reported that semantic knowledge of prototypical and cultural important life events is not altered in patients with schizophrenia (16). Hence, one could hypothesize that event models of complex personal events would be preserved in patients with schizophrenia and would play an effective structuring role in their event segmentation, at least when sensory information about these events is available.

### Chronology of the Memory

The present study showed that patients with schizophrenia were as able as controls to chronologically order what they had lived 1 week earlier. It is worth noting that the mean chronological score of both groups (about 7/12) allows us to rule out any ceiling effect explaining our results. Therefore, the absence of significant difference cannot be explained by the easiness of the task. We had hypothesized that an abnormal processing of temporal order may influence patients’ capacity to order complex events in time. However, despite patients’ deficits in elementary temporal processing (highlighted by the temporal order judgment task), both the non-autobiographical stories sequencing task and the memory chronological organization task were well performed by patients. It might indicate that, contrary to the basic temporal order task in which the temporal order judgment is mostly based on the perception of time lapse between two stimuli, the sequencing tasks of more complex stories involve various cognitive and inferential processes together entangled with a chronological representation of the event. Hence, patients could have successfully achieved the sequencing tasks using other cues or information to infer or understand the chronological order.

### Memory Content

In the current study, we observed that patients with schizophrenia retrieved significantly fewer details than control participants during the free recall task. More precisely, the analyses of the narratives, according to Jeunehomme and colleagues’ method (50), showed that fewer experience units were recalled by patients in comparison with controls. Together, these results highlight a quantitative impairment of patients’ memory recall. They confirm previous findings showing that autobiographical memories of patients with schizophrenia are less detailed and less vivid (17, 19,) and also extend them to the memories of recently lived events. It is worth noting here that participants had reviewed event pictures before the free recall task when performing the segmentation task (a prereview that can be considered as an external executive help, enhancing either event re-encoding or event recall). Nevertheless, patients had difficulties to retrieve as many details as controls from the original event. This deficit, observed in spite of executive support, might be considered as an argument in favor of an encoding deficit in those patients. Looking more precisely at the components of experience units recalled, we observed that patients had specifically difficulties recalling actions with interaction, as they reported them twice less than control participants, the other components being in similar proportion in both groups. Actions with interaction correspond to actions performed by the participants themselves. Interestingly, patients with schizophrenia reported fewer of these moments when they were agent of the event, whereas they recalled as well as controls other components of the event such as persons, objects, thoughts, spatial movements, or perceptual details. This result aligns with previous research showing that patients often describe passive connections to others, experience events as imposed upon them by external forces/agents (53, 54), or even define themselves using passive items (28). This phenomenon reflecting a lack of agency (55, 56) may explain patients’ difficulties engaging in peer relationships and social activities and possibly their poorer memory for actions.

### Relationship Between Event Segmentation and Event Memory

Correlation analyses were conducted to better understand the relationship between the segmentation of a personal event and the associated autobiographical memory. Our results showed that in the control group, a smaller number of boundaries, corresponding to larger events, was related to higher performances in the chronological order task. Large events are thought to be related to meaningful units, supporting the event understanding and *in fine* allowing its better memorization (25). Our results showed a relationship, in control participants, between large event segmentation and good performances in the chronological order task. These observations are in line with those of Zacks and collaborators (2006), who showed significant correlations between high performances on event segmentation and a good chronological organization of its memory.

In the patient group, we observed that the higher typicality of the event segmentation was associated with a higher number of experience units recalled. Here again, our results provided evidence of a relationship between event segmentation and autobiographical memory.

### Limitations

Some methodological limitations should be acknowledged. First, although the walk in the city center was thought to match participants’ assumed daily life, this event was particularly salient for patients in comparison with controls. Due to patients’ social withdrawal and impoverished daily life, the outdoor circuit contrasted with their daily life, and this salience might have enhanced memory encoding (57). It would have been relevant to assess participants’ subjective experience of the walk (i.e., personal importance, distinctiveness, motivation) and to control its impact on memory. Second, patients included in the present study displayed less severe psychotic symptoms relatively to those of Zalla’s study (27) and were not impaired in the non-autobiographical stories sequencing tasks contrary to the patients included by Zalla and colleagues. This suggests that our results might not be generalized to all patients with schizophrenia.

### Perspectives and Conclusion

The present study showed that the memory of a recent personal life event was impaired in terms of vividness and actions remembered in patients with schizophrenia, whereas the event segmentation abilities and temporal organization of memory remained preserved in patients. Hence, pictorial material might help structuring the event temporal organization in schizophrenia but is not enough to compensate patients’ recall deficits.

This study calls for further investigations in order to explore other factors that might account for memory deficits in schizophrenia. Indeed, initial perceptual deficits, working memory impairments, or encoding disorder have been thought to explain patients’ memory impairments. A more recent study also pointed out that neurological soft signs might be correlated with autobiographical memory performance (58). Future studies using wearable cameras or virtual reality could provide new insights on the role of those factors, as they enable to control and manipulate the to-be-encoded event.

Despite the methodological limitations inherent to ecological protocols, studies investigating daily life cognition are crucial to grasp patients’ cognitive impairments, better understand their disability, and try to rehabilitate them in their everyday life.

## Data Availability Statement

The datasets generated for this study are available on request to the corresponding author.

## Ethics Statement

The studies involving human participants were reviewed and approved by Strasbourg Hospital Ethic Committee. The patients/participants provided their written informed consent to participate in this study.

## Author Contributions

FB, J-MD, and AG designed the study. MA and JP collected the data. MA, JP, FB, and AG conducted the analyses. NM supervised the method and statistical analyses. MA wrote the first draft of the manuscript. FB provided substantial contributions to the manuscript. All authors read and approved the final version of the manuscript.

## Conflict of Interest

The authors declare that the research was conducted in the absence of any commercial or financial relationships that could be construed as a potential conflict of interest.

## Footnotes

1 For instance, if an event was identified by all participants of the reference group (100%), its associated score was 1; if only 80% of participants had identified the event, its score was 0.8.
